# Long-Term Functional Stability of Organic and Inorganic Modified Luminescent Lyocell Fibers for Security Applications

**DOI:** 10.3390/ma19091767

**Published:** 2026-04-26

**Authors:** Aleksandra Erdman, Jadwiga Gabor, Natalia Brzezińska, Maciej Pyza, Magdalena Popczyk, Piotr Kulpiński, Andrzej S. Swinarew

**Affiliations:** 1 Centre of Papermaking and Printing, Lodz University of Technology, Wólczańska 223, 90-924 Łódź, Poland; aleksandra.erdman@p.lodz.pl; 2 Faculty of Science and Technology, University of Silesia in Katowice, 75 Pułku Piechoty 1A, 41-500 Chorzów, Polandnatalia.brzezinska@us.edu.pl (N.B.); maciej.pyza@us.edu.pl (M.P.); magdalena.popczyk@us.edu.pl (M.P.); 3 Department of Mechanical Engineering, Informatics and Chemistry of Polymer Materials, Faculty of Textiles and Design, Lodz University of Technology, Żeromskiego 116, 90-924 Łódź, Poland; piotr.kulpinski@p.lodz.pl; 4 Institute of Sport Science, The Jerzy Kukuczka Academy of Physical Education, 40-065 Katowice, Poland

**Keywords:** aging tests, lyocell fibers, luminescent fibers, security fibers

## Abstract

Luminescent cellulose-based fibers are promising materials for anti-counterfeiting applications because they can provide covert and spectrally distinguishable optical signatures compatible with paper- and textile-based authentication systems. In this study, Lyocell fibers modified with selected inorganic and organic luminescent compounds were subjected to accelerated xenon-lamp aging in order to evaluate their functional durability under simulated environmental exposure. The effects of aging on the mechanical properties and luminescent behavior of the fibers were investigated. The results showed that accelerated aging led to a reduction in tensile strength and elongation at break for all fiber variants, although the extent of these changes depended on the type of modifier. Spectroscopic analysis indicated that, despite changes in emission intensity, the characteristic luminescent responses of the modified fibers remained detectable after aging. These findings suggest that luminescent Lyocell fibers can retain their practical identification potential under the applied test conditions and may be considered promising candidates for use as covert security elements. The observed stability is attributed to the immobilization of luminophores within the cellulose matrix and the intrinsic photostability of the applied luminescent systems. At the same time, the study highlights the need for further investigations into the structural and photophysical stability of such systems under long-term environmental exposure.

## 1. Introduction

According to the report of the Organisation for Economic Co-operation and Development (OECD) and the European Union Intellectual Property Office (EUIPO), global trade in counterfeit and pirated goods causes substantial economic losses and remains a serious international challenge [1]. Counterfeiting affects not only financial performance and intellectual property protection, but also consumer safety, product authenticity, and brand credibility. Therefore, the development of efficient and difficult-to-reproduce security features remains an important issue in modern materials engineering and industrial practice [2,3,4,5,6,7].

Among the many anti-counterfeiting approaches currently under investigation, luminescent materials are particularly attractive because they can generate specific optical signals under selected excitation conditions while remaining invisible or only weakly visible under ambient light [2,3,4,5,6]. Such systems can be used in banknotes, security documents, product labels, luxury packaging, and textile-based identification elements. In recent years, optical nanomaterials, rare-earth-doped phosphors, upconversion systems, aggregation-induced emission luminogens, and room-temperature phosphorescent materials have all been extensively explored for information encryption and anti-counterfeiting applications due to their tunable emission properties and potential for multi-level authentication [2,3,4,5,6,7].

In this context, cellulose-based luminescent materials are of particular interest because cellulose is abundant, renewable, biodegradable, and compatible with a wide range of paper and fiber-based products [7,8,9,10]. In addition to its structural role, cellulose may also serve as a host matrix or immobilizing environment for luminescent compounds, enabling the preparation of functional materials with optical identification properties [7,8,9]. This makes cellulose especially attractive for anti-counterfeiting systems intended for substrates such as paper, nonwovens, and fibrous composites.

Previous studies have demonstrated that regenerated cellulose fibers can be successfully modified with luminescent inorganic compounds, including lanthanide-doped nanomaterials and oxide- or fluoride-based phosphors [11,12,13,14,15,16]. Such fibers may be used as covert optical markers and can be incorporated into paper products or textile structures [14,15]. In addition to inorganic modifiers, organic luminescent compounds have also attracted considerable interest as potential optical additives due to their tunable spectral properties and molecular design flexibility [17,18,19].

Among regenerated cellulose materials, Lyocell fibers produced by the N-methylmorpholine-N-oxide (NMMO) process are of particular importance. In contrast to conventional viscose technology, the Lyocell process enables direct cellulose dissolution without derivatization and is considered a more environmentally favorable route for the preparation of regenerated cellulose products. Furthermore, the dry–wet spinning method used in this process provides a convenient route for introducing functional additives directly into the spinning dope, which enables the preparation of cellulose fibers with advanced optical or application-specific properties [20,21].

For practical implementation, however, the usefulness of luminescent security fibers depends not only on their initial emission properties, but also on their long-term stability under environmental exposure. In real application conditions, such materials may be subjected to solar radiation, ultraviolet light, temperature fluctuations, humidity, and oxygen, all of which may affect both the polymeric substrate and the embedded luminescent systems [22,23]. These factors may lead to changes in mechanical integrity, optical response, or overall functionality, which is especially important in the case of anti-counterfeiting materials expected to maintain their identification performance during storage and use.

Accelerated aging tests are commonly used to simulate the effects of environmental exposure within a shortened experimental period and to estimate the durability of functional materials under service-like conditions [22,23,24,25,26,27]. Such methods have been widely applied to polymer composites, structural materials, and optical systems [22,23,24,25], and they are also relevant for cellulose-based functional materials intended for long-term use. In the case of luminescent systems, aging is particularly important because irradiation and photo-oxidative processes may lead not only to intensity loss, but also to spectral changes or degradation of the emitting species, which may compromise the practical detectability of the luminescent signature [28].

Although numerous studies have described the preparation and optical performance of luminescent materials for anti-counterfeiting applications [2,3,4,5,6,7], considerably less attention has been paid to the long-term functional stability of luminescent regenerated cellulose fibers under accelerated environmental aging. This issue is especially relevant when such materials are intended for practical authentication systems, where retention of a recognizable luminescent response may be more important than absolute emission intensity alone.

The modifiers selected in this work do not constitute a chemically uniform family of luminophores, but instead represent a practically relevant group of optically distinct luminescent systems compatible with cellulose spinning. Accordingly, the objective of the present study is not to compare their intrinsic photophysical efficiencies, but to determine whether chemically different emitters retain identifiable emission signatures after accelerated aging when permanently embedded in a Lyocell fiber structure.

Therefore, this study investigates the effect of accelerated xenon-lamp aging on the mechanical and luminescent properties of Lyocell fibers modified with selected inorganic and organic luminescent compounds. Particular attention is given to the retention of characteristic emission profiles, as these spectral signatures are of primary importance for authentication and anti-counterfeiting applications. In addition to changes in luminescent behavior, the study also evaluates the mechanical response of the modified fibers after aging in order to assess their functional durability under simulated environmental exposure.

**Figure 1:**
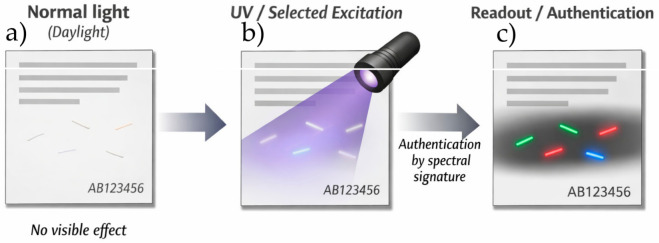
Conceptual illustration of phosphor-modified Lyocell fibers as covert security markers in paper or textile substrates: (**a**) no visible effect under ambient light, (**b**) activation under UV or selected excitation, and (**c**) authentication based on characteristic emission signatures.

The novelty of the present work lies in the comparative evaluation of long-term functional stability in a series of luminescent Lyocell fibers containing chemically different classes of emitters introduced directly into the cellulose spinning dope (Figure 1). The significance of the study lies in determining whether the luminescent identity of such fibers can be preserved after accelerated weathering, which is essential for their potential use as covert security elements in paper-based and textile-based authentication systems.

## 2. Materials and Methods

To produce cellulose fibers modified with luminescent additives, PLACETAE pulp from Rayonier^®^ (Rayonier, Wildlight, FL, USA) was used. The pulp was characterized by an α-cellulose content of 98.4%, a moisture content of 5.90%, and a degree of polymerization (DP) of 1236. The cellulose solvent employed was a 50% aqueous solution of N-methylmorpholine N-oxide (NMMO) (Huntsman Corporation Belgium NV, Everberg, Belgium). The solvent was a clear, amber, suspension-free fluid with a maximum conductivity of 200 µS/cm and a maximum light absorption of 0.25 at 450 nm (1 cm path length). The NMMO content was 49–51%, N-methylmorpholine (NMM) content ≤ 1%, and other amines ≤ 0.5%. The solution had a viscosity of 7 cP at 20 °C and a freezing point of −20 °C.

To stabilize the molecular weight of cellulose during spinning dope preparation, gallic acid propyl ester (Tenox PG, Sigma^®^, St. Louis, MO, USA) was used. Tenox PG is a white powder with a molar mass of 212.2 g/mol and a purity of ≥98%.

### 2.1. Inorganic Luminescent Compounds for Fiber Modification

For the preparation of luminescent fibers, the following inorganic compounds, synthesized and characterized at the Rare Earth Department of Adam Mickiewicz University in Poznan, Poland, were used:

- CeF_3_:5%Tb.

The CeF_3_:5%Tb fluoride was obtained via a precipitation reaction in the presence of glycerin. Lanthanide nitrate solutions served as substrates, and glycerin was used as the reaction medium. The synthesis was conducted under a protective argon atmosphere. The resulting white suspension was centrifuged and repeatedly rinsed with distilled water. The obtained paste was subsequently used to modify cellulose fibers. Further details regarding this compound are provided in [11].

- YOF:5%Eu.

In order to obtain Eu^3+^ doped oxy fluoride, the Pechini sol–gel method was used with yttrium Y (NO_3_)_3_ and europium Eu(NO_3_)_3_ nitrates. In addition, the firing temperature of the gel precursors was increased to 700 °C. Details of the synthesis and the compound’s characteristics are presented in the article [12].

- CeF3:15%Tb.

This compound was synthesized using the same procedure as CeF3:5%Tb, as described in [11].

- Sr_2_CeO_4_.

Cerium–strontium oxide was prepared via the Pechini sol–gel method using strontium nitrate (Sr(NO_3_)_2_) and cerium(III) chloride (CeCl_3_) as substrates. The gel precursors were fired at 900 °C. Detailed synthesis procedures and compound characteristics are described in [13].

Detailed procedures for the synthesis of the inorganic phosphors used in this study have been described in our previous works [11,12,13]. Therefore, only a brief description is provided here.

### 2.2. Organic Luminescent Compounds for Fiber Modification

Two groups of organic compounds, obtained by research teams from the Department of Polymer Chemistry and the Department of Biomaterials at the University of Silesia in Katowice, Poland, were used to modify the fibers. The characteristics of the organic luminescent compounds applied for the modification are presented below.

**Figure 2:**
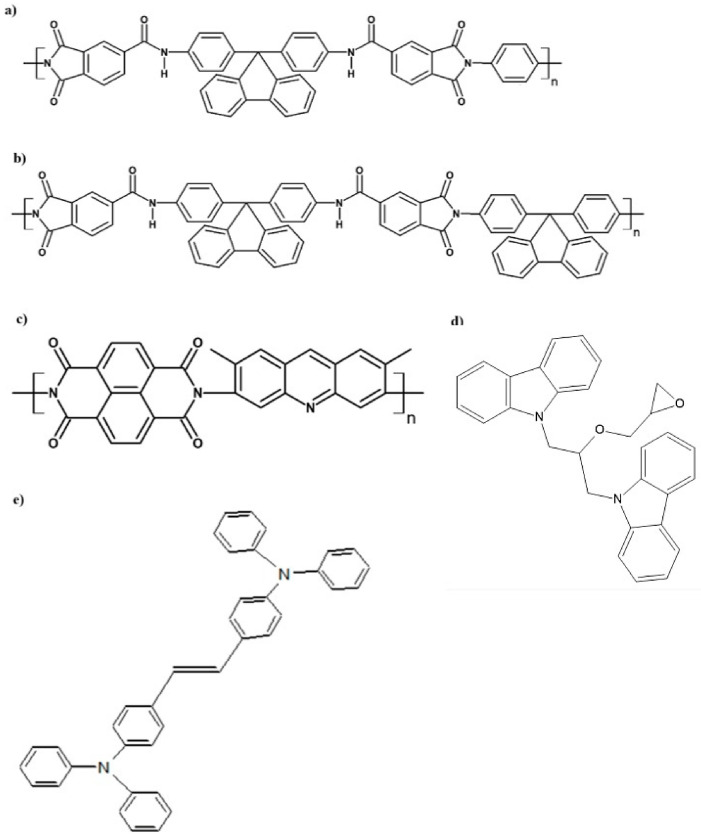
Chemical structure of the (**a**) S-48 polymer; (**b**) S-49 polymer; (**c**) GR-125 polymer; (**d**) AS10; (**e**) AS11.

The first group of compounds, developed at the Department of Polymer Chemistry, consisted of polyamidoimides containing 9,9-diphenylfluorene groups. The chemical structures of the individual polymers are shown in Figure 2a–c, while their characteristics and optical properties are described in [14]. Another organic luminescent modifier was a polyimide containing acridine yellow moieties, described in [15], whose chemical structure is presented in Figure 2d.

Luminescent organic compounds produced at the Department of Biomaterials of the University of Silesia in Katowice are also included; their chemical structures are shown in Figure 2e.

### 2.3. Spinning Dope Preparation

Cellulose dissolution in an aqueous NMMO solution was carried out using a laboratory kneader (IKA VISC type MKD 0.6-H60, Staufen, Germany) with a working capacity of 300 mL. The spinning solutions were prepared by introducing appropriate amounts of shredded pulp, 50% aqueous NMMO solution, Tenox stabilizer, and a selected luminescent modifier into the kneader. The proportions of the components were selected to obtain 240 g of the final spinning solution with a cellulose concentration of 8% and a water content of about 15%.

The dissolution process was conducted for 1.5 h, during which the temperature was gradually increased to 112 °C under constant stirring and reduced pressure (240 hPa). During processing, excess water was removed from the system to achieve the appropriate component ratio required for cellulose dissolution in NMMO. The process was controlled by continuous monitoring of temperature, vacuum level, and the volume of water distilled from the system.

The concentrations of luminescent modifiers used are presented in Table 1. A concentration of 0.5 wt% was selected for rare-earth modifiers to ensure sufficient functional activity while maintaining acceptable processability of the spinning dope. In contrast, polymer modifiers were limited to 0.1 wt% due to their strong influence on solution rheology and fiber formation, where even low concentrations are effective, and higher loadings may adversely affect spinnability and structural uniformity.

### 2.4. Fiber Formation

Cellulose fibers were formed using the dry–wet spinning method with a laboratory spinner equipped with an 18-hole spinneret (channel diameter 0.4 mm, length 3.5 mm). The spinning solution prepared as described above was placed in a piston spinning head and extruded at 115 °C at a constant rate. The piston speed was adjusted to obtain a solution flow velocity through the spinneret channels of 1 m/min.

After extrusion, the streams of spinning solution passed through a 10 cm air gap and then entered a coagulation water bath maintained at 20 °C. The residual solvent was removed from the solidified fiber bundle in a rinsing bath at 85 °C. The obtained fibers were dried at room temperature without applied tension.

### 2.5. Aging Tests

Accelerated aging tests were carried out at the Central Institute for Labour Protection in Łódź using a Xenotest 150 S+ aging chamber (Atlas, Mount Prospect, IL, USA). The tests were conducted in accordance with PN-EN ISO 105-B04 [29] (weather resistance) and PN-EN ISO 105-B02 [30] (light resistance) standards.

Samples for aging were prepared by applying and fixing a thin layer of fibers onto plastic plates measuring 13 cm × 4 cm. The prepared samples were mounted in dedicated frames and placed in the chamber. Fiber samples were exposed to irradiation and water spray in two cycles of 100 h each. Irradiation was performed using a xenon arc lamp with a color temperature of 5500–6500 K. Water spraying was applied cyclically (1 min spraying followed by 29 min drying).

After each cycle, the mechanical and luminescent properties of the fibers were evaluated and compared with those of non-aged reference samples.

### 2.6. Determination of Mechanical Properties

To evaluate the effect of artificial aging on mechanical performance, tensile strength parameters were measured for fibers before aging and after the first and second aging cycles. Basic tensile properties were determined in accordance with PN-EN ISO 5079:1999 [31].

Mechanical testing was performed using a Zwick Z 2.5 universal testing machine (Ulm, Germany) equipped with computer control. The testXpert v. 7.1 software was used to control the testing procedure, record measurement data, and perform calculations and statistical analysis. Fiber strength was determined by measuring the force acting on filaments stretched at a constant rate.

### 2.7. Luminescent Properties of Modified Cellulose Fibers

When a phosphor is excited by monochromatic radiation, the intensity of emitted light depends on the excitation wavelength. This relationship is represented by emission spectra. Conversely, excitation spectra illustrate the dependence of emission intensity at a fixed wavelength on the varying excitation wavelength. Excitation spectra were recorded at the wavelength corresponding to maximum emission, while emission spectra were recorded at the wavelength corresponding to maximum excitation.

Luminescent properties were measured using a FluoroMax-4 spectrofluorimeter (Horiba Jobin Yvon, Edison, NJ, USA) and a Hitachi F-7000 fluorescence spectrophotometer (Hitachi, Tokyo, Japan). Fiber samples were introduced into the instruments as bundles of parallel fibers, and measurements were performed without mounting plates. Emission and excitation spectra were obtained for selected phosphor-modified fibers before aging and after the first and second cycles of accelerated aging. The excitation wavelengths were selected based on the maxima of the excitation spectra for each system, taking into account the specific absorption mechanisms of the luminophores (e.g., 4f–5d transitions, 4f–4f transitions, or π–π* transitions), to ensure optimal and selective emission detection.

### 2.8. Visualization of the Embedding of Modifiers

**Figure 3:**
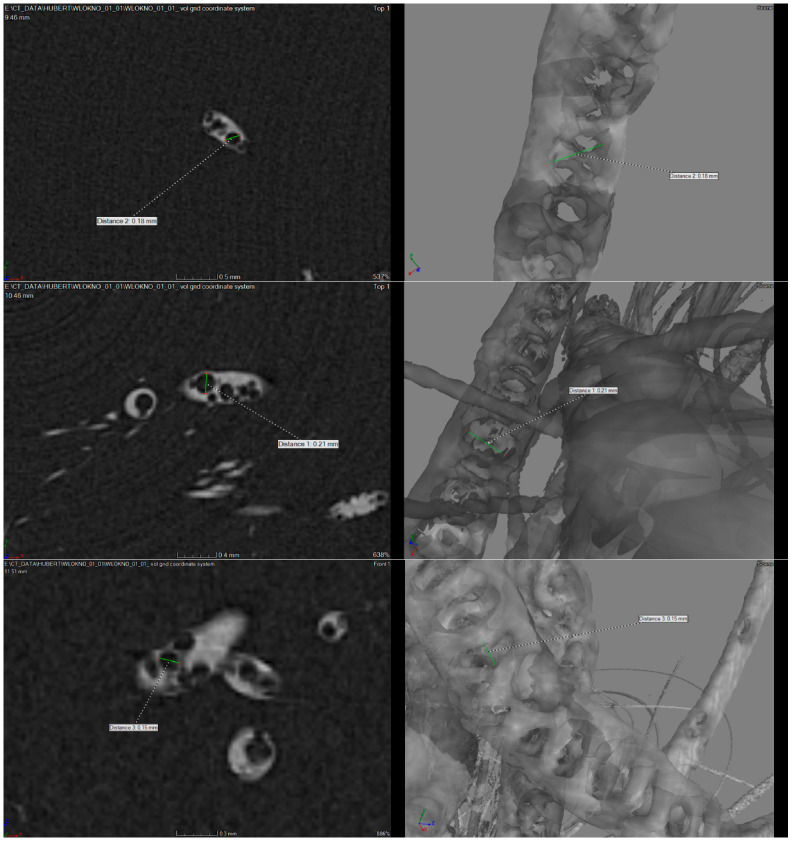
High-resolution X-ray scans revealing the internal structure of the fibers and confirming the location of the modifiers.

Structural analysis was performed for fiber B3 with AS11 organic modifier to determine the spatial distribution of modifiers within the fiber structure. The study revealed the presence of voids within the internal fiber structure, confirming the incorporation and localization of the modifiers (Figure 3).

A two-tube high-resolution X-ray scanner (GE Phoenix v|tome|x, Boston, MA, USA) equipped with a DXR250 detector (GE, Boston, MA, USA) was used, enabling image acquisition at a resolution of 2024 × 2024 pixels. For each sample, 2000 projections were recorded with a total scan time of 30 min at an accelerating voltage of 150 kV and a current of 100 µA. These parameters allowed acquisition of images with optimal contrast and a spatial resolution of 30 µm.

After scanning, the two-dimensional projections were reconstructed using Phoenix datos|x CT 2.0 software (GE, Boston, MA, USA). Three-dimensional visualizations were generated using VGStudio 2.1 (Volume Graphics, Nagoya, Japan).

## 3. Results and Discussion

The mechanical behavior of the modified fibers is strongly influenced by the dispersion and distribution of the luminescent modifiers within the cellulose matrix. Although detailed microstructural characterization (e.g., SEM and EDX analysis) is not included in the present study, such analyses have been previously reported for analogous systems and confirmed uniform dispersion of both inorganic and organic luminophores within regenerated cellulose fibers.

In particular, earlier studies demonstrated that the applied modification approach enables effective incorporation of luminescent particles without the formation of large agglomerates, resulting in relatively homogeneous fiber structures. Therefore, the observed differences in mechanical properties between samples (e.g., increase in toughness for sample B6 and decrease for sample B3) may be attributed to differences in modifier–matrix interactions and local structural effects rather than to macroscopic inhomogeneity [11,17,32,33,34].

To determine the effect of artificial aging on fiber properties, selected fibers containing the same amount of modifier in the fiber matrix were analyzed. For fibers containing inorganic luminescent compounds, the modifier concentration was 0.5 wt%, whereas fibers containing organic modifiers contained 0.1 wt%. The results of mechanical and luminescent tests for the selected fibers are presented below.

### 3.1. Mechanical Properties of Fibers Modified with Luminescent Compounds After Aging

**Figure 4:**
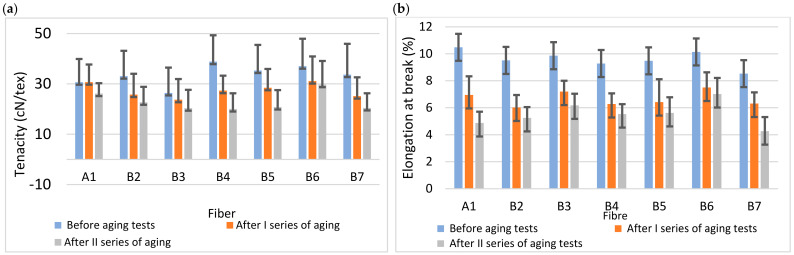
(**a**) Tenacity at break before and after accelerated aging; (**b**) elongation at break before and after accelerated aging.

The effects of accelerated aging on the mechanical properties of fibers modified with luminescent organic compounds are summarized in Table 2 and Table 3 and illustrated in Figure 4a,b.

Accelerated aging caused deterioration of the mechanical properties of fibers modified with organic luminescent compounds. The observed changes were comparable to those recorded for fibers modified with inorganic phosphors. When interpreting the results, it should be noted that the samples were introduced into the testing apparatus as layers of parallel fibers. Their arrangement may have slightly influenced the measured mechanical parameters after aging. Based on the obtained results, 200 h of exposure reduced the tenacity of fibers modified with inorganic additives by approximately 28%, while fibers containing organic modifiers exhibited a decrease of about 29%. Elongation at break decreased by approximately 41% for fibers modified with inorganic compounds and by about 40% for fibers containing luminescent polymers, relative to the values measured before aging.

The reduction in mechanical strength observed after accelerated aging is consistent with the known susceptibility of cellulose materials to photochemical degradation. Exposure to UV radiation may induce chain scission reactions within the cellulose backbone, resulting in a decrease in the degree of polymerization and consequently reduced tensile strength and elongation at break. Similar behavior has been reported for other cellulose-based and biopolymer materials subjected to artificial weathering conditions. Importantly, the decrease in mechanical parameters was comparable for fibers containing inorganic phosphors and those modified with organic luminescent polymers. This observation suggests that the presence of luminescent modifiers at the applied concentrations (0.5 wt% for inorganic and 0.1 wt% for organic additives) does not significantly accelerate degradation of the cellulose matrix. Instead, the mechanical deterioration appears to be primarily governed by environmental exposure affecting the regenerated cellulose itself.

The observed differences in absolute mechanical strength do not contradict this conclusion, as the analysis is based on relative degradation after aging, which remains comparable for both modified and unmodified fibers.

From an application perspective, this level of mechanical degradation does not necessarily limit the use of such fibers in anti-counterfeiting systems. Security fibers embedded in paper or textile matrices typically serve as identification markers rather than structural load-bearing elements. Therefore, preservation of optical properties is more critical than maintaining the original mechanical strength.

### 3.2. Luminescent Properties Before and After Accelerated Aging

**Figure 5:**
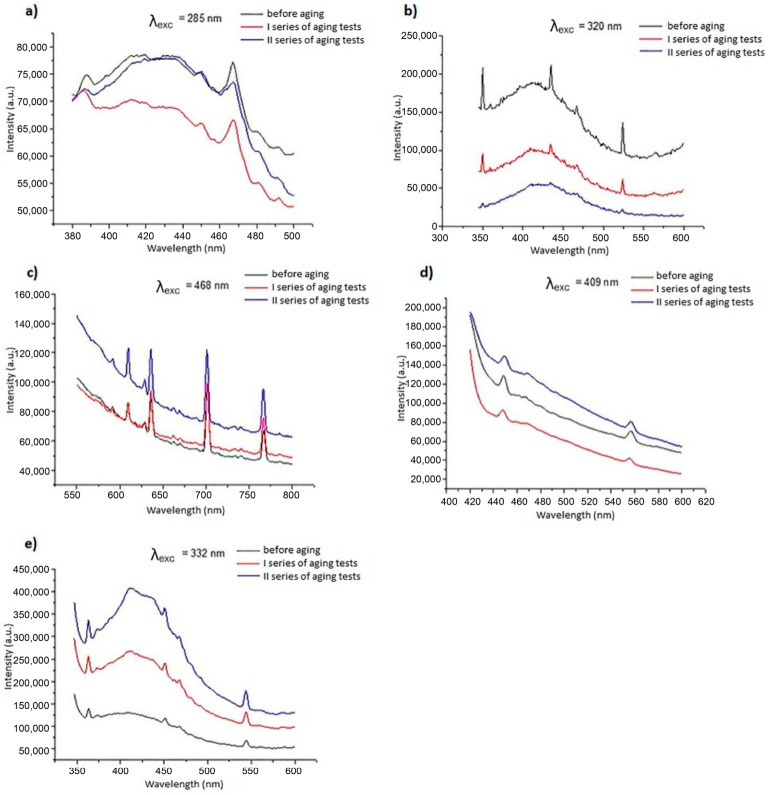
Emission spectra of fibers modified with inorganic luminescent compounds, (**a**) A1, (**b**) A2, (**c**) A3, (**d**) A4, (**e**) A5.

**Figure 6:**
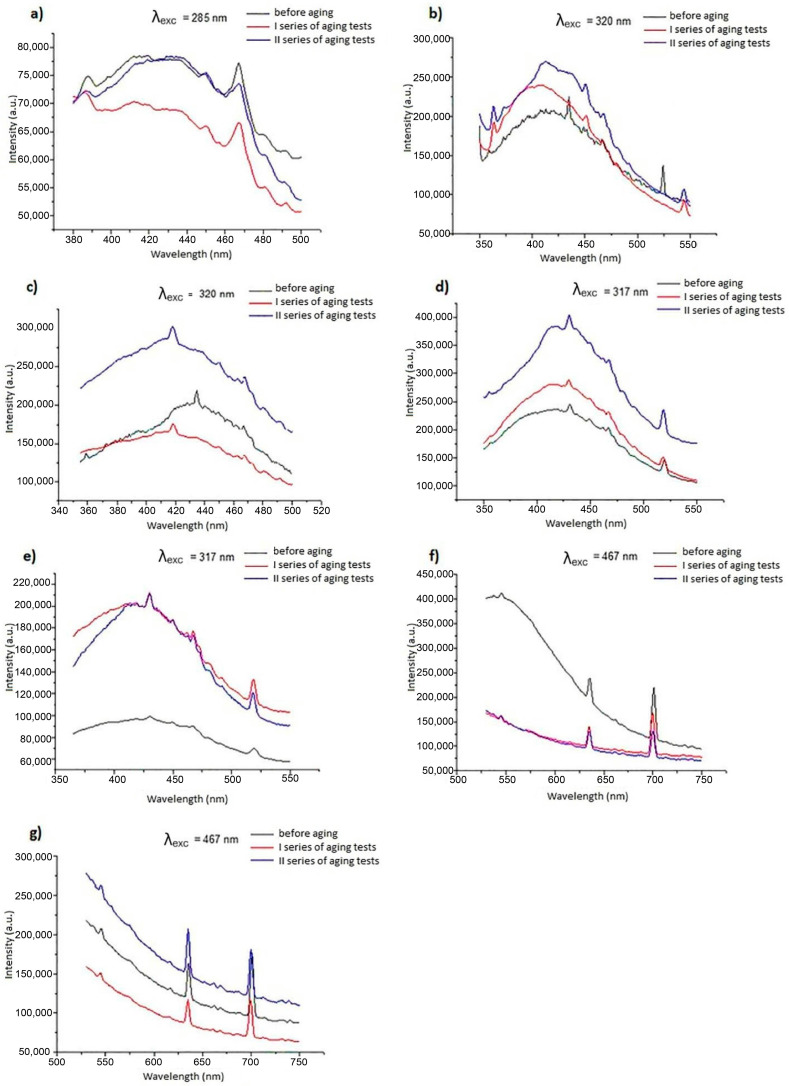
Emission spectra of fibers modified with organic luminescent compounds, (**a**) A1, (**b**) B2, (**c**) B3, (**d**) B4, (**e**) B5, (**f**) B6, (**g**) B7.

The results of studies evaluating the effect of artificial aging on luminescent properties are presented in Figure 5 and Figure 6. To facilitate interpretation of the emission spectra shown in Figure 5 and Figure 6, the main emission bands were assigned to the corresponding electronic transitions of the applied luminophores.

For fibers modified with inorganic phosphors, the observed bands are consistent with characteristic rare-earth transitions. In CeF_3_:Tb^3+^ systems (A2, A4), the dominant emission at ~545 nm is attributed to the ^5^D_4_ → ^7^F_5_ transition of Tb^3+^, accompanied by weaker bands corresponding to ^5^D_4_ → ^7^F_6_ (~490 nm), ^5^D_4_ → ^7^F_4_ (~585 nm), and ^5^D_4_ → ^7^F_3_ (~620 nm). The band at ~350 nm is associated with Ce^3+^ 4f–5d transitions acting as a sensitizer. For YOF:Eu^3+^ (A3), emission arises from Eu^3+^ transitions, with the main peaks at ~590 nm (^5^D_0_ → ^7^F_1_) and ~612–615 nm (^5^D_0_ → ^7^F_2_). In Sr_2_CeO_4_ (A5), the broad band in the 400–500 nm range is attributed to O^2−^ → Ce^4+^ charge-transfer transitions. The emission observed for the reference fiber (A1) in the 420–440 nm range is related to intrinsic cellulose fluorescence.

For organic modifiers (B2–B7), emission bands located mainly in the 400–500 nm region are assigned to π–π* transitions within conjugated systems, with possible contribution of intramolecular charge transfer effects. Observed spectral shifts and band modifications after aging can be attributed to changes in conjugation and partial photooxidative processes.

These assignments confirm that, despite intensity variations, the characteristic emission mechanisms and spectral signatures remain preserved after accelerated aging.

Due to the fibrous form of the materials, an identical geometric arrangement of samples during successive measurements was not possible. Therefore, the analysis focuses on spectral shape and band position rather than absolute intensity, as these parameters are more reliable for assessing functional luminescent stability in this type of material, while detailed photophysical characterization (e.g., quantum yield or decay time) is beyond the scope of this application-oriented study.

This results in differences in absolute emission intensity across spectra and does not indicate structural changes in the material. Instead, the appearance or disappearance of bands and changes in relative band intensity provide meaningful information about material behavior.

The following spectral changes were observed after aging:

- **A1**—gradual decrease in the 430–440 nm band relative to the 410–420 nm band.
- **A2**—gradual fading of signals at 351 nm, 436 nm, and 524 nm.
- **A3**—no significant spectral changes.
- **A4**—formation of a distinct band with a maximum at 468 nm.
- **A5**—strong increase in the 375–430 nm band.
- **B2**—strong increase in the 375–430 nm band.
- **B3**—shift in the maximum from 433 nm to 417 nm and smoothing of the 430–414 nm band.
- **B4**—formation of a band in the 400–420 nm range.
- **B5**—formation of a signal with a maximum at 430 nm.
- **B6**—band fading up to 620 nm.
- **B7**—no significant spectral changes.

These results indicate diverse aging effects, suggesting differences in modifier resistance and, particularly in the 430–440 nm region (characteristic for unmodified cellulose), variations in the resistance of the cellulose matrix itself. In both modifier groups, some fibers (A3 and B7) showed no significant spectral changes. Luminescent properties were preserved after 200 h of aging. No major spectral changes indicative of loss of luminescent identity were observed under the applied test conditions. Quantitative determination of luminescence intensity changes using spectrofluorometric methods is difficult and poorly reproducible; therefore, unequivocal evaluation of intensity changes was not possible. However, the absence of significant spectral shape changes indicates that luminescent performance will be maintained under service conditions. From an application perspective, the spectral profile is the key functional feature when such fibers are incorporated into paper or textile materials. The differences observed between individual fibers can be attributed to the distinct photophysical mechanisms governing luminescence in inorganic phosphors and organic luminophores. Inorganic phosphors based on rare-earth ions are generally characterized by high photostability due to the shielding of 4f electronic states by outer electron shells. As a result, their emission bands are typically resistant to photochemical degradation. This may explain the relatively stable spectral profiles observed for fibers containing compounds such as CeF_3_:Tb or YOF:Eu. Organic luminescent polymers, in contrast, may be more susceptible to photochemical processes such as photo-oxidation, conformational changes, or partial degradation of conjugated structures. However, the results obtained in this study indicate that embedding these compounds within the cellulose matrix provides a certain degree of protection against environmental exposure. The matrix may act as a physical barrier limiting oxygen diffusion and reducing direct exposure of luminophores to UV radiation. An important observation is that although some changes in emission intensity and minor spectral shifts were detected, the overall spectral signatures remained identifiable after aging. In anti-counterfeiting applications, the spectral fingerprint rather than absolute intensity is typically the primary authentication parameter. Therefore, preservation of the spectral profile indicates that the functional performance of the luminescent fibers is largely retained. The absence of significant spectral shifts also indicates that the corresponding CIE chromaticity coordinates are expected to remain largely unchanged after aging.

### 3.3. Correlation Between Artificial Aging Time and Natural Exposure

Relating artificial aging duration to natural exposure conditions is not straightforward. In the present study, a Xenotest 150 S+ apparatus was used, in which the radiation intensity inside the chamber cannot be directly controlled. Therefore, the relationship between artificial aging and natural exposure was estimated using data from the Atlas aging test guide and the PN-EN ISO 105-B02 standard.

The conversion between artificial aging time and natural exposure can be calculated using:

H=E×3.6×t

where

*H*—radiation dose (kJ/m^2^);

*E*—irradiance (W/m^2^);

*t*—exposure time (h);

3.6—conversion factor.

According to the standard, irradiance during testing should be 42 W/m^2^. Therefore, for 200 h exposure:

$$H=42\times 3.6\times 200=30240\;\mathrm{kJ}/m^{2}$$

To estimate equivalent natural exposure, annual UV radiation data for Florida (2800 kJ/m^2^, 295–385 nm) were used:

2800 kJ/m^2^ → 1 year;

30,240 kJ/m^2^ → x years.

x≈10.8 years

Thus, 200 h of artificial aging corresponds to an approximate UV dose equivalent on the order of one decade of outdoor exposure under high-irradiance conditions, but this should not be interpreted as a direct service-life prediction.

This value should be treated as indicative. Artificial xenon lamp radiation approximates natural sunlight but is not identical. Additionally, latitude, atmospheric conditions, temperature, and humidity influence material degradation. Therefore, the calculations presented provide a preliminary estimate of the durability of luminescent cellulose fibers.

### 3.4. Proposed Mechanism of Luminescent Stability in Modified Cellulose Fibers

The obtained results suggest that the long-term stability of the luminescent properties is closely related to the structural role of the regenerated cellulose matrix. During the NMMO dry–wet spinning process, luminescent modifiers are incorporated directly into the spinning dope and become physically embedded within the cellulose network during fiber formation. As a consequence, the luminophores are immobilized inside the regenerated cellulose structure rather than being deposited only on the fiber surface. The cellulose matrix may therefore act as a protective environment, partially shielding the luminescent compounds from direct exposure to ultraviolet radiation, oxygen, and moisture during environmental aging. This effect is particularly important for organic luminophores, which are typically more susceptible to photochemical degradation than inorganic rare-earth phosphors. This stability results from the combined effect of physical immobilization of luminophores within the cellulose matrix, reduced exposure to environmental quenchers, and, in the case of inorganic phosphors, the intrinsic photostability of shielded 4f electronic transitions. The spatial confinement of the modifiers within the fiber structure may limit diffusion of reactive species and reduce the probability of photochemical reactions leading to structural decomposition. At the same time, the observed deterioration of mechanical properties indicates that the cellulose matrix itself undergoes partial degradation during accelerated aging, most likely due to UV-induced chain scission and oxidation processes typical for cellulose materials. However, despite this structural weakening, the embedded luminescent compounds retain their characteristic emission bands. This suggests that the optical functionality of the fibers is primarily governed by the intrinsic stability of the luminophores combined with their immobilization within the cellulose matrix.

## 4. Conclusions

Accelerated xenon aging revealed that luminescent Lyocell fibers retain their spectral emission characteristics despite prolonged environmental simulation. While mechanical properties decreased by approximately 40%, the observed degradation is not critical for their intended function as security markers, where structural load-bearing capacity is secondary to optical performance. The absence of major spectral shifts suggests that both organic and inorganic phosphors remain structurally stable within the cellulose matrix. The results suggest that embedding within the regenerated cellulose structure may contribute to preserving the identifiable emission profile during accelerated aging. Although UV dose calculations suggest an equivalent service life of approximately ten years, this estimation should be interpreted cautiously, as natural aging involves additional oxidative and environmental factors not fully replicated in accelerated testing. Overall, phosphor-modified cellulose fibers demonstrate promising long-term functional stability for anti-counterfeiting applications in documents, labels, and textiles. These findings contribute to the understanding of the long-term behavior of luminescent cellulose fibers and provide important guidance for the design of durable fiber-based security elements in advanced anti-counterfeiting systems.

## Figures and Tables

**Table 1:** Concentration of modifier in spinning dope solutions.

Fiber	Modifier	Concentration in Spinning Dope (wt%)
A1	-	0
A2	CeF_3_:5%Tb	0.5
A3	YOF:5%Eu	0.5
A4	CeF_3_:15%Tb	0.5
A5	Sr_2_CeO_4_	0.5
B2	AS10	0.1
B3	AS11	0.1
B4	S48	0.1
B5	S49	0.1
B6	KB43	0.1
B7	GR125	0.1

**Table 2:** Comparison of tenacity before and after aging of selected fibers modified with organic luminescent compounds.

Fiber	Modifier	Tenacity Before Aging Tests (cN/tex)	Standard Deviation	Tenacity After I Series of Aging (cN/tex)	Standard Deviation	Tenacity After II Series of Aging (cN/tex)	Standard Deviation
A1	-	30.65	9.27	30.69	7.0	26.17	4.11
B2	AS10	33.09	10.05	25.76	8.27	22.7	6.14
B3	AS11	26.41	10.08	23.72	8.21	20.34	7.32
B4	S48	38.84	10.49	27.33	5.93	20.03	6.25
B5	S49	35.32	10.16	28.45	7.49	20.82	6.73
B6	KB43	37.05	10.91	31.13	9.79	29.71	9.4
B7	GR125	33.7	12.22	25.15	7.47	20.46	5.8

**Table 3:** Comparison of elongation at break before and after aging of selected fibers modified with organic luminescent compounds.

Fiber	Modifier	Elongation at Break Before Aging Tests (%)	Standard Deviation	Elongation at Break After I Series of Aging Tests (%)	Standard Deviation	Elongation at Break After II Series of Aging Tests (%)	Standard Deviation
A1	-	10.48	1.62	6.95	1.38	4.87	0.84
B2	AS10	9.51	1.34	6.02	0.93	5.25	0.8
B3	AS11	9.86	1.41	7.2	0.8	6.18	0.86
B4	S48	9.28	1.39	6.28	0.79	5.53	0.74
B5	S49	9.48	1.12	6.42	1.7	5.62	1.16
B6	KB43	10.14	1.19	7.50	1.13	7.02	1.19
B7	GR125	8.53	0.92	6.31	0.83	4.27	1.04

## Data Availability

The original contributions presented in this study are included in the article. Further inquiries can be directed to the corresponding author.
